# Students’ Online Information Searching Strategies and Their Creative Question Generation: The Moderating Effect of Their Need for Cognitive Closure

**DOI:** 10.3389/fpsyg.2022.877061

**Published:** 2022-05-11

**Authors:** Shibo Mao, Di Wang, Chaoying Tang, Pinhua Dong

**Affiliations:** ^1^School of Economics and Management, University of Chinese Academy of Sciences, Beijing, China; ^2^Educational Research Central of Primary and Secondary School of Chaoyang District, Beijing, China; ^3^MOE Social Science Laboratory of Digital Economic Forecasts and Policy Simulation, University of Chinese Academy of Sciences, Beijing, China

**Keywords:** creative question generation, online information searching, need for cognitive closure, middle school students, creativity

## Abstract

With the wide application of computers and digital technologies, online information searching is being integrated into students’ learning process. Improving students’ creative question generation through online information searching is an emerging research topic in the creativity and pedagogy field. Online information searching brings diversified information, but it also leads to cognitive load brought by a large amount of online information. Using online information searching to generate creative questions depends on students’ cognitive properties. However, the existing literature ignores the joint influence of students’ online information searching strategies and cognitive properties on their creative question generation. This study puts forward three hypotheses: first, the two strategies of students’ online information searching (“keywords” and “Web page exploration”) will increase their creative question generation; second, the impact of “keywords” is negatively moderated by students’ need for cognitive closure (NFCC); third, the impact of “Web page exploration” is positively moderated by NFCC. The main reason is that high NFCC prevents students from obtaining diversified perspectives by using different keywords, but it helps to avoid distractions caused by a large amount of online information and promote the persistency of their reading information. Based on the data of quasi-experimental tasks completed by 90 students in Grade 7 and Grade 8, the results support the above hypothesis. The contributions of creative question generation theory and NFCC theory, as well as important issues of future study, are discussed.

## Introduction

### Information Searching and Creative Question Generation

The ability to generate creative questions is important to students’ creativity (Craft, 2001), which is also of great significance in the field of pedagogy. For example, asking good questions is a critical task in scientific courses (Shepardson and Pizzini, 1991; Chin and Osborne, 2008), which can motivate students’ learning, critical thinking, and achievements (Yu et al., 2015). Creative question generation is also regarded as an important index of creativity (Laius and Rannikmae, 2003).

The combination of remote knowledge is a primary factor for creativity (Ward, 2008). With the wide application of computer and digital technology, the Internet has become an important information source and assistant for students’ creativity (Tang, 2014; Tang and Ding, 2014). Students’ online information searching strategies would potentially influence their creative question generation. A related study is about online information searching and problem-solving performance (Oloruntegbe et al., 2010). They found that information searching is a necessary step of problem solving. For example, to finish ill-structured tasks, information browsing, scanning, learning, and recognition are needed (Belkin et al., 1993; Ellis and Haugan, 1997). Evidence suggests that extensive searches for information and quick scans for information (Brand-Gruwel et al., 2009), use of relevant keywords, selection of appropriate information sources, and extraction of important content will lead to more successful web-based problem solving (Hwang and Kuo, 2011).

In line with the theory of three levels of the cognitive behaviors (Kitchner, 1983), Glaubman et al. (1997) pointed out that qProblems can also be divided into three levels, including surface problems that rely on memory, problems that require comprehension, and complex problems that are based on higher-order thinking. To ask a question, students need to identify the key elements of the question, the relationship between the elements, and the goal to be achieved (English, 1997). During the process of questing, students need to reflect on the information received, and refine and transform the information into its personally meaningful form (Bangert-Drowns et al., 2004). Generating creative questions is an “ill-structured” work characterized by low verifiability, vague standards, and a lack of absolute definition (Chi et al., 1981). It needs abilities of information browsing, thinking, integration, identifying and clarifying alternative opinions, positions, and perspectives (Jonassen, 1997), and “revising and integrating” [which means part of the text will gain more attention and linkages among sections will be figured out, Rouet et al. (2001)]. That is, the search for online information for the purpose of generating creative questions is a cognitive process (Kitchner, 1983). The positive side of the abundance of online information is that it will bring a variety of information or perspectives that will benefit students’ creativity. However, with distracting online information, students will generate cognitive load, which will lead to cognitive inhibition and reduce students’ creativity. It is likely that the effect of online information searching on the generation of creative questions will be influenced by students’ need for cognitive closure (NFCC). NFCC is an epistemic motivational tendency to seize and freeze on ideas that come to mind early and easily (Webster and Kruglanski, 1994). NFCC consists of four facets: preference for order, predictability, intolerance of ambiguity, and close-mindedness. It is likely that the effect of information searching strategies on question posting will be moderated by students’ NFCC. High NFCC students have the tendency of choosing an adherent solution rather than seeking out alternative solutions or exploring diverse perspectives (Chirumbolo et al., 2004) and preferring to conform to the dominant norms (Manchi Chao et al., 2010). This will reduce the likelihood that students with high NFCC will gain diverse perspectives on the Internet by using different keywords. Therefore, high NFCC reduces the contribution of keyword search strategies to students’ creative question generation. On the other hand, because a lot of online information will cause cognitive load and inhibit higher-order thinking, thus it will be detrimental to creativity. However, students with high NFCC tend to make decisions quickly, so they are less distracted. Furthermore, it was found that, when people with high NFCC are told that uncreative ideas are not the target, they will exert more effort to persistently search for alternative solutions to find their perceived normative creative solutions (Ong and Leung, 2013). This will motivate them to read more information and access more diverse information online. As such, their browsing of more online pages will help them generate creative questions.

So far, the co-influence of information searching strategies and cognitive properties on creative question generation is neglected by scholars. To fill this gap, the study will introduce NFCC into a relationship between online information searching strategies and creative question generation. The findings of this study will contribute to question generation and students’ creativity theory in the digital world.

The structure of the rest of the paper includes: the second part is literature review and hypotheses generation; the third part is the introduction of quasi-experimental design; The fourth part is the data analysis steps and the results; and the last part is to discuss the theoretical contribution, limitations of this study, and research issues for future studies.

## Literature Review and Hypotheses

### Creative Question Generation

Creative question generation (question finding, question posting, and question asking) refers to forming and raising questions in the process of thinking activities. According to literature in the creativity research field, creative question generation is positively correlated with diverse and flexible thinking (English, 1997; Brown and Walter, 2005). The ability to ask creative questions is regarded as an index of students’ creativity (Laius and Rannikmae, 2003). The main evaluation methods include Torrance Tests of Creative Thinking (TTCT), in which the participants are asked to post questions as much as possible for the given pictures. The outputs are evaluated in terms of originality, fluency, etc. (Torrance, 1966). Another evaluation method is to ask students generate questions about their school or daily life, and analyze the number and novelty of their questions (Runco and Okuda, 1988). In pedagogy, students’ question-generation ability was positively correlated with their learning achievements in courses (Yu et al., 2015). Generating questions is used as an instructional method to motivate students’ critical thinking, hypothesis building, and analysis of diverse online sources. There is evidence that when the students began their research project with an open question instead of a research topic, the quality, depth, and breadth of the research and writing improved dramatically (Haefeli, 2016). Taken together, fostering students’ ability of creative question generation will stimulate the development of their creativity and learning.

According to Glaubman et al. (1997), the quality of questioning can be divided into three levels: low-level questions, which are practical and surface level, ask for an individual’s memorization; medium-level questions ask for the ability of understanding; and more integrative and complicated high-level questions ask for in-depth reasoning, analysis, synthesis, and evaluation. In order to generate high-level or creative questions, higher-order thinking skills are required. English (1997) points out that the cognitive task of asking questions mainly includes determining the key factors of the problem and how they are related to each other and the goal to be achieved. During the process of generating questions, students need to reflect on the information received and elaborate and transform the information into personally meaningful forms (Bangert-Drowns et al., 2004). The process is affected by the habitual state of constructing personal knowledge and employing various cognitive and metacognitive strategies (Yu and Liu, 2009). Taken together, generating creative questions requires information, knowledge, and higher-order thinking.

### Online Information Searching Behaviors and Creative Question Generation

With the wide application of computer and digital technology, students can easily obtain rich information on the Internet. It was found that searching professional knowledge from the Internet enhanced students’ creativity (Tang, 2014; Tang and Ding, 2014). Students’ online information access depends on their strategies of searching information. Scholars pointed out the seven strategies of online information searching: “number of keywords,” “maximum depth of exploration,” “total number of web page exploration,” “revisited web pages,” “total time for browsing the result pages,” “total time for browsing web pages for less than 7 s,” and “total time for browsing web pages for more than 7 s” (Tsai et al., 2012). They can be measured by analyzing the recording of students’ information searching behaviors (Hwang et al., 2008; Thatcher, 2008; Tu et al., 2008). For instance, Tu et al. (2008) recorded students’ online searching behaviors by a screen capture software called “Camtasia Recorder.”

Among them, using keywords is a widely used strategy of searching online information (Hwang and Kuo, 2011). Keywords are the basic components of concepts and relationships. Using appropriate keywords in information searching positively predicted eighth graders’ (14-year-olds) web searching performance (Tu et al., 2008). “Number of keywords” means the number of different keywords used in searching information on the Internet. When searching with different keywords on the Internet, a variety of online information will be browsed, which will lead to a broad thinking space for students? As such, students will be more likely to gain diversified information. Considering that there is a close relationship between external diversified information and creativity (Tang and Ye, 2015), “number of keywords” will benefit students’ creative question generation.

We also suggest that another frequently used strategy “number of Web page exploration” will benefit creative question generation. One reason is that it brings the chance to read more online information. In addition, according to the Dual-Path theory of creativity (Nijstad et al., 2010), which argues that creativity—the generation of original and appropriate ideas—is a function of cognitive flexibility and cognitive persistence, and that dispositional or situational variables may influence creativity either through their effects on flexibility, on persistence, or both. In this study, “number of web page exploration” stands for the cognitive persistence students spent for completing the task which, in turn, benefits their creativity. Hence, we put forward the hypotheses as below:

Hypothesis 1: “number of keywords” as students’ online information searching strategy will positively impact their creative question generation.

Hypothesis 2: “number of web page exploration” as students’ online information searching strategy will positively impact their creative question generation.

### Need for Cognitive Closure and Creative Questioning Generation

The NFCC is an epistemic motivational tendency to seize and freeze on ideas that come to mind early and easily. Individuals with high NFCC tend to oppose uncertainty, ambiguity, or confusion (Webster and Kruglanski, 1994). NFCC consists of four facets: preference for order, predictability, intolerance of ambiguity, and close-mindedness. NFCC is associated with a lower creative capability (Ip et al., 2006). Because high-NFCC individuals rely more on conventionalized norms and established knowledge, and prefer to believe that their chosen solutions are correct rather than seek out alternative solutions or explore diverse perspectives. Thus, their ideational fluidity is restricted and which, in turn, leads to fewer ideas (Chirumbolo et al., 2004).

The NFCC is likely to influence students’ online information searching process. The theory of information searching as learning points out that searching not only satisfies the need of getting information but also brings forth learning outcomes because the searching process is accompanied by iterative, reflective, and integrative sessions that facilitate critical and creative learning (Rieh et al., 2016). Considering the close relation between NFCC and learning outputs, the effectiveness of the cognitive process of online information searching would be influenced by the students’ NFCC.

It was found that individuals with high (vs. low) NFCC would select more decision-supportive information and less decision-challenging information for viewing (Hart et al., 2012). Compared to low NFCC, high NFCC individuals need a smaller amount of information to make a final choice (Choi et al., 2008). In addition, high NFCC would be more likely to conform to the dominant norms (Manchi Chao et al., 2010). As such, high NFCC prevents students from taking full value from diversified knowledge searched by the strategy of “numbers of keywords” and thus decreases their creative question generation. Hence, we point out the following hypothesis:

Hypothesis 3: NFCC will moderate the relationship between students’ “number of keywords” and their creative question generation, that is, high NFCC students will find it more difficult to generate creative questions by using the strategy of “number of keywords.”

On the other hand, NFCC also has a positive effect on creativity (Ong and Leung, 2013) and creative question generation. According to Treisman’s (1964) Attenuation theory, students’ attention to online information will be selective in order to prevent the information processing system from becoming overloaded (McLeod, 2008). Students of high NFCC tend to make quick decisions and prefer the perception of epistemic security vs. remaining in a state of uncertainty. Their heuristics decision-making styles ask for simple cues and readily available information (Roets et al., 2015), and are less likely to consult more information (Hiel and Mervielde, 2002). So they are likely to shut out irrelevant distractions and noise, and their cognitive activities will demand less cognitive resources (Chirumbolo et al., 2004). As such, students of high NFCC are likely to avoid cognitive load caused by the large amount of online information searched by “number of web page exploration.” Moreover, compared with low NFCC, people with high NFCC cognizant realized that uncreative ideas are consensually invalid solutions; they will exert more effort to persistently search for alternative solutions to find their perceived normatively prescribed creative solutions (Ong and Leung, 2013). The persistence pathway of creativity can generate creative solutions through hard work and constant systematic probing of alternative ideas (Nijstad et al., 2010). Online information from different web pages is often controversial, which may arouse their persistency. Hence, we put forward the following hypothesis:

Hypothesis 4: NFCC will moderate the relationship between students’ “number of web page exploration” and their creative question generation, that is, high NFCC students will more likely generate creative questions by using the strategy of “number of web page exploration.”

## Materials and Methods

### Participants

The participants of this study were 90 students belonging to five classes of Grade 7 and Grade 8 in a public middle school located in Beijing, China with a good reputation for its education quality. The school’s principal agreed to help us in this academic study. She then invited the two teachers of the Information Technology course to join us. They were informed that it was an academic study; all the data would be presented anonymously. All data on students, such as their names and scores, will be kept confidential and converted into code. The quasi-experimental activity will be completed in computer rooms of the middle school.

In selecting the students’ grades, their computer operation skills are an important consideration due to their influence on the performance of Internet searching (Lazonder et al., 2000; Tu et al., 2008). Therefore, the participants would be able to search information on the Internet, and their computer operation ability would be similar. After discussing with the principal and the teachers, five classes of Grade 7 and Grade 8 students were selected. These students all had gained several basic computer courses, and their computer operation skills can satisfy the requirement of the experimental activity. In addition, their teachers introduced that their computer operation skills were similar according to their performance in the computer courses. Therefore, no observable difference of their computer skills was expected in this study.

A total of 90 students were selected to participate in this study. Among them, 35 were boys, 38.9% and 55 were girls, 61.1%. In total, 2.2%, were 11 years old, 42.2% were 12 years old, 50% were 13 years old, 4.4% were 14 years old, and 1.1% were 15 years old.

### Experimental Task and Process

The activity was carried out in their self-study class in the afternoon when they had finished the days’ course learning; it lasted for three afternoons. The whole process of the quasi-experiment lasted 40 min. The process of activities was as follows: students of each class came into the computer room of the school. All the computers there had been installed with the screen capture software named “Screen Video Experts” by researchers in earlier days. Thus, each student’s information searching behaviors on the Internet were recorded by “Screen Video Experts.” In the computer room, there were two researchers and a teacher. In the first 10 min, the teacher presented to the whole class that the purpose of this study was to practice creative question generation through using the computer to search information on the Internet. They were instructed and demoed the detailed steps of opening the software “Screen Video Expert.” They were given 15 min to complete the task: “Please search information in the Internet to raise a creative question related with terracotta warriors and horses with less than 100 words.” The task was co-designed by three middle school teachers and researchers of this study. The task was believed appropriate to be completed by students of Grade 7 and Grade 8. During the process, no other instruction was offered, and discussion was not allowed. After finishing the task, the students were asked to fill out a questionnaire with items of NFCC, their demographical information, and other variables not used in this study. The detailed quasi-experimental process is reported in Supplementary Appendix 1.

### Measures

#### Online Information Searching Strategies: Number of Keywords, Number of Web Page Exploration

The online information searching strategies were measured by analyzing the video of a 15-min task completing the process for each student. The evaluation method came from previous studies (Lin and Tsai, 2007; Tu et al., 2008; Tsai et al., 2012). Two researchers of this study watched the video and evaluated the number of different keywords and web page independently. If their data were different, they co-observed them and got the right score.

#### Need for Cognitive Closure

The NFCC was measured by the short form of NFCC generated by Roets and Van Hiel (2011) based on Webster and Kruglanski (1994). The English instrument was converted to Mandarin Chinese, using a back-translation procedure (Brislin, 1970). Sample items include: “I don’t like situations that are uncertain,” “I dislike questions that could be answered in many different ways.” Cronbach’s *a* in this study is 0.771.

#### Creative Question Generation

Consensus assessment technique (CAT, Amabile, 1982) was used to measure the students’ ability to generate a creative question. CAT is appropriate for measuring creative outputs. For the student who failed to figure out a creative question, the score was “0.” Otherwise, the score was “1.” To satisfy the requirements of CAT, the answers of the students were evaluated by a master’s degree student, a Ph.D. student, and an associate professor in the organizational behaviors field. They had a half-hour training by a researcher in this study before their scoring. They evaluated independently, and their Cronbach’s *a* was above 0.90. For several questions that got different scores, they discussed face-to-face to come to an agreement. The detailed examples of rating are reported in Supplementary Appendix 2.

#### Control Variables

The students’ age, gender, grade, and class were controlled to prevent their gender difference (Hawkins and Power, 1999), class difference, and age difference in asking questions. In addition, their academic scores in Chinese, Mathematics, and English in the last semester and their other abilities, such as reading ability of online information and writing ability of the question, were controlled.

## Results

Pearson correlations between NFCC and the two strategies show that NFCC had no significant correlation with the two variables. The “number of keywords” positively correlated with the “number of web page exploration” (*r* = *0.513, p* < *0.01*). For details, see Table 1. The score difference of “number of keywords” and “number of web page exploration” between the group that successfully generated a creative question and the group that failed to generate a creative question was examined. The results showed that there were no significant differences between them (*t* = 0.359, *p* = 0.723; *t* = −0.457, *p* = 0.649, respectively).

**TABLE 1:** Correlations.

Main variables	1	2	3	4	5	6
1. Need for cognitive closure	1					
2. Number of keywords	0.039	1				
3. Number of web page exploration	0.022	0.513**	1			
4. Chinese	0.280*	−0.075	−0.013	1		
5. Math	0.281*	−0.039	0.128	0.298**	1	
6. English	0.210	−0.029	−0.022	0.470	0.668	1
Mean	3.745	3.00	3.22	86.01	93.02	96.39
SD	0.822	1.635	1.901	6.770	7.146	3.636

*p < 0.05, **p < 0.01, two-tailed test.

Since the dependent variable is a binary variable, the errors need to obey the binomial distribution. The maximum likelihood method is appropriate to be used to estimate the regression coefficient. The formula of the logistic regression model is as below:


(1) $$L\,o\,g\,i\,t\,\left(P\right)=b_{0}+{\text{b}}_{0}\,{\text{x}}_{0}+\cdots +{\text{b}}_{\text{p}}\,{\text{x}}_{\text{p}}$$

Where, b_i_ (i = 1, …, p) represents the regression coefficient of each variable.

The formula of the logistic regression is as below:


(2) Logit⁢(P)=Ln⁢[P/(1-P)]

P/(1-P) stands for the occurrence ratio (odds), that is, the ratio of the probability of occurrence at a certain time to the probability of non-occurrence. Based on the calculation of equation (2), the equation of P is as below:


(3) $$P=\left[Exp\left(b_{0}+{\text{b}}_{0}{\text{x}}_{0}+\cdots +{\text{b}}_{\text{p}}{\text{x}}_{\text{p}}\right)\right]/\left[1+Exp\left(b_{0}+{\text{b}}_{0}{\text{x}}_{0}+\cdots +{\text{b}}_{\text{p}}{\text{x}}_{\text{p}}\right)\right]$$

Where, Exp (b_i_) means the odds ratio (OR).

The three steps of the logistic regression were as follows: (see Table 2) first, all control variables were put into the model, and then, the NFCC, number of keywords, and number of web page exploration. Finally, their two interactive items were entered into the model. It turned out that the χ2 of OMNIBUS test of the interactive regression model was 21.155, *df* = 12, *p* = 0.048, which means the model is significant or had a predictive effect. The −2 logarithmic likelihood value of the first model with constant variables was 83.032, and when all the predictor variables entered the model, the value significantly decreased to 68.146. Meanwhile, the χ2 of Hosmer-Lemeshow test is 10.665 with a *p*-value above 0.05 (*p* = 0.221); thus, the goodness-of-fit indices were acceptable.

**TABLE 2:** Logistic regressions.

Variable	B	S.E.	P	Exp(B)	EXP(B)95% CI	
					Low	High
Gender	0.255	0.615	0.678	1.290	0.387	4.303
Age	−1.178	1.074	0.273	0.308	0.038	2.526
Class	−0.325	0.300	0.279	0.723	0.402	1.300
Grade	0.721	1.724	0.676	2.056	0.070	60.348
Chinese score	0.040	0.059	0.498	1.041	0.927	1.168
Math score	−0.058	0.059	0.327	0.944	0.841	1.059
English score	0.023	0.110	0.831	1.024	0.825	1.270
NFCC	0.152	0.402	0.704	1.165	0.530	2.559
Number of keywords	0.156	0.210	0.456	1.169	0.775	1.763
Number of web page exploration	−0.109	0.195	0.574	0.896	0.612	1.313
Number of keywords *NFCC	−1.249	0.511	0.014	0.287	0.105	0.781
Number of web page exploration *NFCC	1.285	0.422	0.002	3.614	1.581	8.261

*p < 0.10.

The regression results showed that gender, age, class, grade, and students’ academic scores of Chinese, Math, and English, all insignificantly influenced the possibility of generating creative questions.

The number of keywords and number of web page exploration had no significant impact on the possibility of generating a creative question [β = 0.156, *p* = 0.456, *OR* (95%) = 1.169 (0.775–1.763); β = −0.109, *p* = 0.574, *OR* (95%) = 0.896 (0.612–1.313), respectively]. Thus, Hypotheses 1 and 2 are rejected. We also found that the NFCC failed to influence the possibility of generating a creative question [β = 0.152, *p* = 0.704, *OR* (95%) = 1.165 (0.530–2.559)].

Moreover, the interactive items of the number of keywords and NFCC negatively predicted the possibility of generating a creative question [β = −1.249, *p* = 0.014, *OR* (95%) = 0.287 (0.105–0.781)]. This means that every 1% increase in both the number of keywords and NFCC will reduce the possibility of raising new questions by.287 times. Moreover, the interactive item of “number of web page exploration” and NFCC positively predicted the possibility of generating a creative question: β = 1.285, *p* = 0.002, *OR* (95%) = 3.614 (1.581–8.261). This means that every 1% increase in both the number of web page exploration and NFCC will improve the possibility of generating a creative question by 3.614 times. Thus, Hypotheses 3 and 4 are supported.

## Discussion

This study uses the screen-capturing method to collect two kinds of online information searching strategies of middle school students and analyze their impact on creative question generation. NFCC is introduced into the model to see the more nuanced effect of online information searching on creative question generation.

The main findings of this study are: first, NFCC negatively moderates the relationship between the strategy of “number of keywords” and the students’ creative question generation. Our explanations are that the narrow attention and selective reading tendency of high NFCC students prevent them from taking different perspectives from the information searched by numbers of keywords. It, in turn, decreased their generation of creative questions. This finding keeps in line with the previous literature on the relationship between NFCC and information searching (Li and Liu, 2013). As such, this study demonstrated that online information searching is a cognitive process, and the outputs of the process depend on cognitive properties.

Second, NFCC negatively moderates the relationship between the strategy of “number of web page exploration” and students’ creative question generation. Our explanations are that the preference of making quick decisions and the heuristic decision-making style of high NFCC students helps them have less trouble with the large amount of online information. Therefore, the cognitive load they encounter when browsing and reading web pages will be reduced. Meanwhile, high NFCC students tend to adhere to the social norm, and the controversy of online information might motivate them to persist in reading the information on the web pages. Thus, they will have more chances to access different pieces of online information, which benefits their creative question generation. To be noted, unlike the previous study, which regarded NFCC as a temporary cognitive characteristic, which was manipulated by increasing cognitive load (Ford and Kruglanski, 1995), the NFCC in this study reflects students’ cognitive properties, which are more stable compared with manipulated NFCC. The finding of this study keeps in line with the previous conclusion that high NFCC is less distracted (Wu et al., 2020) and more selective in processing information (Kardes et al., 2004). A similar result came from an experimental study, that is, in the high cognitive loading task condition, high NFCC individuals had less negative priming effect. That is, high-NFCC people are able to deal with distractors more efficiently than low-NFCC people when they are under cognitive load (Kossowska, 2007). Thus, the positive side of NFCC on creativity should not be neglected.

Third, this study did not find that “number of keywords” improved creative question generation. This seems to conflict with a previous study that found that ill-structured problems require solvers to use more keywords for searching alternative views or perspectives and explore different keywords that help overcome functional fixedness (Nijstad et al., 2010). Our explanation is that, in this quasi-experimental study, the “number of keywords” does not equal diversified keywords. It is hard to accurately rate the diversity of keywords just based on the language of keywords because the categorization of keywords should depend on the students’ implicit understanding of the words.

Fourth, this study did not find that the “number of web page exploration” improved creative question generation. The strategy relates to how much the potential new information is accessed. This seems to conflict with a previous conclusion that external diversified knowledge contributes to creativity (Tang and Ye, 2015). Our explanation is that, in this study, students were asked to finish the task within 15 min so they would face the time stress of generating creative questions. Meanwhile, the large amount of information from the web pages brought a cognitive load of reading and understanding which, in turn, made their cognitive load even worse. This inhibited the contribution of a number of web pages to creative question generation.

Taken together, our study suggests that online information searching strategies will co-affect with students’ cognitive processes or properties (albeit NFCC in this study) in raising a creative question. The main theoretical contributions of this study are as follows: first, it contributes to creative question generation by demonstrating that it is influenced by students’ NFCC and their online information searching strategies. With the Internet becoming an important source of information in the digital era, searching information on the Internet has been frequently used as a kind of learning activity in classroom practices. The Internet is becoming a potential assistant to students’ creative question generation. So far, our learning about online information searching strategies is less comprehensive. Scholars have generated a self-reported measurement method of implicit strategies of online information searching, called “Online Information Searching Strategies Inventory” (OISSI, Tsai et al., 2012). It consists of three categories and seven indicators: behavioral (control and disorientation), procedural (trial and error; and problem-solving), and metacognitive (purposeful thinking, selection of main ideas, and evaluation). However, there is no sufficient evidence to support that they are good predictors of online information searching outputs (Çevik, 2015). Except for a study on high school students’ online reading strategies that found that students’ meaning-making, coordinated with self-monitoring and source evaluation, positively influenced their quality of the generated questions, while information-locating strategies contributed little to the participants’ question generation (Cho et al., 2017). In this study, we also collected the data and found they had no significant influence on creative question generation. It may be due to the social-appraisal effect of the self-reporting method, or the students’ lack of sufficient awareness of their strategies. It also might be due to the content it measured; the items include both searching behaviors and the cognitive process. But the main reason we believe comes from the fact that the searching strategy itself fails to predict searching outputs, and cognitive properties should not be neglected.

Second, this study contributes to the NFCC theory by demonstrating its different influences on students’ creative question generation. Extant literature of NFCC seldom considers its effect on information searching (Kardes et al., 2004; Kossowska, 2007; Wu et al., 2020), especially in pedagogy. Meanwhile, few pieces of literature addressed its positive effect on learning. The positive and negative effects found in this study suggest that the four facets of NFCC should have different effects on information searching. Thus, the finding of this study gives a more comprehensive understanding of NFCC and thus enriches NFCC theory.

## Limitations and Future Directions

There are several limitations in this study. First, the students came from a middle school in China. Hence, we should be cautious in generalizing the findings to students of different ages and from other regions. Second, the students’ experience of web searching (Beaufils, 2000; Bilal, 2002) and their internet self-efficacy (Tsai and Tsai, 2003), which will influence their online searching outcomes were not controlled in the model and may bias the results of this study. Third, online information searching might be influenced by the holistic thinking style of the Chinese, which is different from the analytical thinking style of Western culture (Ji et al., 2000). There is evidence that the Chinese need more information than Westerners before making a final attribution (Choi et al., 2003). As a result, strategies of online information searching may be different between Westerners and the Chinese. In addition, it was found that cultural norms are cognitive closure providers (Manchi Chao et al., 2010); students with high NFCC in this study might be more likely to conform to the collectivism of Chinese culture. Thus, more studies are required in order to generalize the findings of this study to different cultures. Fourth, the students’ motivation to do the exercise or their interest in the activity may influence their effort of online information searching and their questioning skills may influence their creative question generation. In this study, their motivation for this quasi-experimental activity and ability to generate question were not controlled in the model. In future studies, they should be taken into consideration. Fifth, the quasi-experimental task of this study is related to a historical topic; the ability to generate creative questions of the topic may be different compared with other topics, such as a fantasy topic.

This study is a preliminary analysis of students’ online information searching and their creative question generation. To improve students’ creative question generation and online information searching, the following important research issues can be explored in the future:

The first research issue is to figure out the key abilities of creative question generation. The widely used originality and fluency of the TTCT test aims to assess the individuals’ creativity based on the evaluation of their creative outputs, which is less suggestive for instructing students’ questioning ability in specific domains. In order to practice question generation as a learning strategy in a classroom, we should know more about the students’ key cognitive abilities and learning skills in the process of creative question generation. The relative research findings will open the black box of creative question generation. To be noted, the concept spaces, systematic components, and the task of completing approaches to generating creative questions may be different from that of finding creative solutions.

The second issue is to explore the relationship between the creative question generation and its required information. Although the Internet has been introduced into the learning practice, we learn less about how to effectively search online information. For example, students often face the challenge of defining their online search needs (Chung and Neuman, 2007). Generating creative questions in different courses (such as math, language, or physics) may ask for different knowledge according to the domain-specific theory of creativity. However, the structure and types of their needed information might be similar, such as the fact, opinion, controversy, and perspective. Research on this issue will shed light on finding the appropriate online information searching strategies and clarify their contribution to creative question generation.

Third, new research methods need to be applied to reflect the coupling between the learning process and the information search process. In extant literature, verbal reports synchronized with screen recordings have been used (Cho et al., 2017); however, it may disrupt the participants’ cognitive process. The neuroscience method may break this limitation and clarify the co-occurrence of searching behaviors and the learning process.

The last but not the least, the four facets of NFCC may have different effects on online information searching outputs. The need for deciding quickly, intolerance ambiguity, and adherence to norms may belong to distinguished psychological characteristics (Kossowska, 2007) and have different effects on information searching. Studying the four facets separately will distinguish the different influence mechanisms of NFCC to online information searching outputs.

## Data Availability Statement

The raw data supporting the conclusions of this article will be made available by the authors, without undue reservation.

## Ethics Statement

The studies involving human participants were reviewed and approved by School of Economics and Management, University of Chinese Academy of Sciences. Written informed consent to participate in this study was provided by their teachers in this study.

## Author Contributions

SM designed the study, analyzed the data, and prepared the draft. DW analyzed the data and revised the writing. CT designed the study, revised the writing, and analyzed the data. PD collected the data. All authors contributed to the article and approved the submitted version.

## Conflict of Interest

The authors declare that the research was conducted in the absence of any commercial or financial relationships that could be construed as a potential conflict of interest.

## Publisher’s Note

All claims expressed in this article are solely those of the authors and do not necessarily represent those of their affiliated organizations, or those of the publisher, the editors and the reviewers. Any product that may be evaluated in this article, or claim that may be made by its manufacturer, is not guaranteed or endorsed by the publisher.
