# Supplementary material for: Students’ Online Information Searching Strategies and Their Creative Question Generation: The Moderating Effect of Their Need for Cognitive Closure
Source: Front Psychol. 2022 May 11;13:877061. doi: 10.3389/fpsyg.2022.877061 (PMC9131088; doi:10.3389/fpsyg.2022.877061)
Supplement: Supplementary file 1 [file Data_Sheet_1.docx]

**Appendix 1: Experimental process**

Step 1. Install the screen capture software named ‘Screen Video Experts’ in all the computers of

The computer rooms by researchers. It can record each student’s information searching behaviors on the Internet.

Step 2: Students of each class enter the computer room and turn on the computers, it shows:

“Welcome and thank you for participating in this academic activity about information searching on the Internet and creative question generation. In the activity, you will complete to generate a creative question after 15 minutes’ information searching on the Internet. At last, before you leave the classroom, you will answer a short questionnaire. All your personal data will keep confidential to your teachers and other outsiders. They will be analyzed anonymous.”

Step 3. Software training (15minutes)

Teacher said: “Before the activity, let me tell you the operating steps of the software we need in the activity.”

- “You will see a chart of on-screen video experts on the desktop. Double click to open it (if prompted, choose Cancel), wait for a few seconds, and then click the trial button at the bottom right.”

- “In the open dialog box, you can modify the file name at the top left and check (only check) below it to record cursor, video and transparent form at the same time.”

-“Click the square icon with a red dot in the upper left corner to start recording. When the answer is finished and stopped, press F3 on the keyboard to return to the current screen, and then click the square icon with a solid box at the top left. Now let's try to record first to see if there is a problem with your computer. If you don't understand anything, please raise up your hand and ask questions.”

Step 4. Finish experimental task (15 minutes)

-Students search information on Internet to generate a creative question.

Step 5. Fill in the questionnaire (10 minutes)

-“Now, you need to save your answers in a doc file with the name "computer number + name.”

-“Then fill a questionnaire about your personal characteristics. Please read the questions carefully, answer them according to your real understanding about yourself. There is no better or worse answer. You don't need to think them for long time, just write down your first reaction.”

**Appendix 2: Example of creative question scoring**

**“0”: Failed to figure out a creative question. The following are four typical types of “0”**

**score questions:**

1. Students only presented some information or fact (with less sentences or many sentences), and didn’t point out any question. For example, “Qin Dynasty is a very important dynasty in China's history. It ended the situation of splitting and separatist regimes for 500 years since the spring and Autumn period, and became the first unified, multi-ethnic and centralized feudal country in China's history. From the reign of the first emperor of Qin to the birth of the third emperor of Qin, the three emperors were handed down and enjoyed the country for 15 years. In 213 BC, Qin Shihuang stipulated that all history books except the historical records of the state of Qin should be burned; In addition to the books on medicine, divination, and tree planting, all the other poems, books and hundreds of languages were burned, which was called "burning books and pitching Confucianism" in history. After the unification of China, Qin took a series of measures to consolidate its rule, including the famous unified currency, unified writing and unified weights and measures.”
2. Students wrote down an unrelative question of the task. For example: “One thing we can't forget is that Qin Shihuang was the founder of the Great Wall in history. After winning the government and unifying China, Meng Tian was ordered to lead 300000 troops, collect Henan, attack the Huns, and build a city in an all-round way under the condition of unity with the outside world. This shows that his totalitarian politics has a practical need (Huang Renyu, Talking about Chinese history by the Hessian River). What deeds of Qin Shihuang in Chinese history are mentioned in the materials? How does the author view the totalitarian politics of Qin Shihuang?”
3. Students asked readers to figure out a real question, they failed to raise a real question by themselves. For example: “The terracotta warriors and horses are a category of ancient tomb sculptures. Terracotta warriors and horses are sacrificial objects made in the shape of soldiers and horses. Terracotta warriors and horses are known as the world's top ten rare treasures. What do you want to say when you see the magnificent terracotta warriors and horses?”
4. Students ask a surface question without deep thinking. For example: “What are the terracotta warriors and horses? What is it for?”

**“1”: Successfully figured out a creative question. The following are four typical**

**types of “1” score questions:**

1. Students generated creative questions that can only be answered after high-order thinking based on their observation. For example: “Do you know why the colorful terracotta warriors and horses lose their original color?” Another example is: “Xiao Ming, Xiao Hong and Xiao Xiao went to visit the terracotta warriors and horses of the Qin Dynasty. They saw many pottery horses in unit 4 of Pit 2, some of which were well preserved and some were broken. At this time, they saw that some pottery horses had saddles, but some did not. So Xiao Ming said: This horse is so badly broken that it doesn't even have a saddle. Xiao Hong disagreed and said: It's not caused by damage. So they argued. Xiaoxiao was wondering who was right? What are the two kinds of pottery horses? Who do you think is the right?
2. Students collected relevant scientific information and creatively figured out a relative question. For example: “Iron is a more active metal. Buried in the underground soil, it is easy to combine with the air and water in the soil to form iron hydroxide and iron oxide. This process is called corrosion. The mixture of iron hydroxide and iron oxide is loose and porous, and the volume expands about 7 times, which is called rust. The volume expansion leads to soil cracks, which promotes the continuous immersion of water and gas sources; Due to the loose rust, it is convenient for the metal iron to continuously contact the oxidation and corrosion of water and gas. In such a vicious cycle, over time, the metal iron will no longer exist, and the rust molecules will slowly diffuse through the cracks of the soil. So, there is no trace of metal and iron. However, iron ions always exist in the surrounding soil in another form. Copper is similar to iron. Can copper also be oxidized?”
3. Students collected relevant historical knowledge and creatively figured out a relative question. For example: “The terracotta warriors and horses located in the pit 1.5km east of the mausoleum of the first emperor of Qin in Lintong District, Xi'an City, Shaanxi Province. The Zhou Dynasty drew lessons from the tyranny of yin and Shang Dynasties and emphasized "Ming De and protecting the people". The birth and implementation of Zhou rites restrained the phenomenon of human sacrifice to a great extent, but it was not eradicated. In the spring and autumn period, countries competed for hegemony, the times were turbulent, and people died again. During the Warring States period, the vassal states successively abolished the system of human sacrifice. In the first year of Qin Xiangong (384 BC), Qin officially abolished the death penalty. Why did the Zhou Dynasty draw lessons from the tyranny of Yin and Shang Dynasties and emphasize "Establishing personality saints and protecting the people"?
4. Students collected relevant information, used their academic knowledge, and creatively figured out a relative question. For example: “From 1974 to 1977, the terracotta warriors and horses pit, one of the burial pits of the Qin Shihuang mausoleum, was excavated one kilometer east of the Qin Shihuang mausoleum. Pit 1 is in the south. It is several meters long and wide from east to west. How many square meters does it cover? Pit 2 is several meters long and wide from east to west, with an area of several square meters. How many square meters is the area of Pit 3? A total of several warrior figurines, several wooden chariots and many pottery horses were unearthed. According to the existing arrangement of terracotta warriors and horses, there may be 7000 terracotta warriors in these three pits, 100 chariots and 100 horses. Pottery servants are tall, usually about 1.8 meters.” Another example: “If you are the son of an archaeologist, your father participated in the excavation of Qin terracotta warriors and horses. He brought back a piece of clay. If you see clay and want to clean it a little, please answer according to the production materials of terracotta warriors and horses. Which of the following methods is desirable and Why: 1. Wipe gently with a small brush; 2. Wash with hot water; 3. Wash with hydrochloric acid”.
